# Microbiome of Apical Intracanal and Extraradicular Biofilms From the Same Roots of Teeth With Persistent Apical Periodontitis: An Observational Study

**DOI:** 10.1111/iej.70113

**Published:** 2026-02-11

**Authors:** Yoko Asahi, Nanako Kuriki, Motoki Okamoto, Daisuke Motooka, Shota Nakamura, Hazuki Maezono, Kittipit Klanliang, Tsuyoshi Shimaoka, Tetsuya Iida, Shigeyuki Ebisu, Yuichiro Noiri, Mikako Hayashi

**Affiliations:** ^1^ Department of Restorative Dentistry and Endodontology, Graduate School of Dentistry The University of Osaka Suita Osaka Japan; ^2^ Research Institute for Microbial Diseases The University of Osaka Suita Osaka Japan; ^3^ Division of Endodontics, Department of Restorative Dentistry and Periodontology, Faculty of Dentistry Chiang Mai University Chiang Mai Thailand; ^4^ Division of Cariology, Operative Dentistry and Endodontics, Department of Oral Health Science Niigata University Graduate School of Medical and Dental Sciences Niigata Japan

**Keywords:** extraradicular biofilm, intracanal biofilm, microbiome, next‐generation sequencing, refractory apical periodontitis

## Abstract

**Aim:**

Bacterial biofilms around the apex are crucial in disease progression and persistence of apical periodontitis. While intracanal biofilms initiate infection, extraradicular biofilms contribute to treatment resistance and persistence. Thus, a comprehensive understanding of these biofilms may help elucidate mechanisms underlying persistent apical periodontitis. Therefore, in this study, we aimed to compare the microbiome and predicted functional profiles in matched apical root canals with those of extraradicular biofilms associated with persistent apical periodontitis.

**Methodology:**

Seventeen root apices from patients with persistent apical periodontitis were collected via surgery. After extraradicular biofilm was collected, intracanal biofilm was obtained by cryopulverisation. Bacterial communities were detected by amplicon sequencing of the V1–V2 region of the 16S rRNA gene. Diversity, microbial composition and predicted bacterial functions were compared between matched intracanal and extraradicular biofilms.

**Results:**

Alpha diversity analysis of the microbiome revealed no significant differences between the two sampling sites. In contrast, the beta diversity of the microbiota of the same root (matched samples) was significantly lower than that of the microbiota of unpaired samples. There were no statistically significant differences in permutational multivariate analysis of variance for the microbiome between paired extraradicular and intracanal biofilms, regardless of the presence of the sinus tract. The abundances of the predominant genera, namely *Fusobacterium*, *Treponema*, *Prevotella*, *Porphyromonas* and *Bacteroides* as well as gram‐positive bacteria, including *Actinomyces*, were similar between extraradicular and intraradicular biofilms. Linear discriminant analysis effect size analysis identified bacterial taxa significantly enriched in extraradicular biofilms, whereas no taxa were significantly enriched in intraradicular biofilms. Phylogenetic Investigation of Communities by Reconstruction of Unobserved States analysis revealed several differences in Kyoto Encyclopaedia of Genes and Genomes pathways between these biofilms.

**Conclusion:**

While comparison of the microbiome between extraradicular and intracanal biofilms of the same root apices revealed differences in bacterial composition, certain similarities were noted, particularly in dominant bacterial species abundance, indicating a close microbial relationship between intracanal and extraradicular biofilms, with some exceptions. Additionally, some differences in predicted functional profiles were observed between the two biofilm types. Thus, the characterisation of bacterial communities around the apical foramen may guide the development of appropriate antimicrobial strategies.

## 1 Introduction

Apical periodontitis is an inflammatory disease primarily resulting from the invasion of oral bacteria into the root canal system and is driven by biofilm formation (Kakehashi et al. 1965; Fukushima et al. 1990; Nair 2006; Ricucci and Siqueira 2010). Numerous studies have focused on identifying root canal bacteria associated with apical periodontitis. Culture‐based studies have identified a range of bacteria, with obligate anaerobes reported to be the most prevalent (Goodman 1977; Sundqvist 1992). Additionally, culture‐independent molecular biology methods have provided further evidence of the relationship between cultivable bacterial species, previously identified by culture‐based techniques, as being associated with apical periodontitis (Sassone et al. 2008). These culture‐independent methods have also identified new bacterial species, including uncultivable ones, as candidate causative agents of apical periodontitis (Siqueira and Rôças 2005; Sakamoto et al. 2008; Rôças et al. 2010).

Advances in next‐generation sequencing have improved our understanding of the microbiota involved in apical periodontitis, revealing a far greater bacterial diversity than previously recognised (Siqueira et al. 2011; Hong et al. 2013; Vengerfeldt et al. 2014). Various studies on root canal microbiota have been conducted, including analysis of microbiota in the root‐filled canal (Siqueira et al. 2016), comparisons of primary and secondary apical periodontitis (Keskin et al. 2017; Bouillaguet et al. 2018), and analysis of the relationship between clinical symptoms and microbiota involved in persistent apical periodontitis (Santos et al. 2011; Sánchez‐Sanhueza et al. 2018; De Brito et al. 2020; Hou et al. 2022). Moreover, a comparative study of the microbiota in matched apical and coronal root segments of infected root canals revealed different bacterial compositions, with a greater abundance of taxa belonging to fastidious obligate anaerobes, including the genera *Porphyromonas* and *Bacteroides*, in the apical segments (Ozok et al. 2012), highlighting different niches between the apical and coronal sides.

Extraradicular biofilms, defined as microbial biofilms attached to the outer root surface near the apex, have been implicated in the refractority and persistence of apical periodontitis (Tronstad et al. 1987; Noiri et al. 2002; Signoretti et al. 2011). Similar to intracanal biofilm microbiota studies, few researchers have also attempted to identify bacteria in extraradicular areas associated with chronic and persistent apical periodontitis (Noguchi et al. 2005; Fujii et al. 2009; Wang et al. 2012; Qian et al. 2019). While many of the detected bacteria were obligate and facultative anaerobes, a wide variation among samples made it difficult to compare the microbiota across different sites with different samples. Histopathological findings indicate that extraradicular biofilms are usually associated with intraradicular biofilms (Ricucci and Siqueira 2010), suggesting a correlation in bacterial composition between extraradicular and intraradicular biofilms. Some studies have reported microbial differences between the root canal and extraradicular area within the same sample. For instance, comparisons of bacterial profiles from root ends, which include both intracanal and extraradicular biofilms and their surrounding periradicular lesions, were conducted using cloning and 16S rRNA sequencing (Subramanian and Mickel 2009) and qualitative and quantitative PCR (Pereira et al. 2017). Bacterial and human proteins from periapical lesions and matched apical intracanal infections were analysed using metaproteome analysis (Provenzano et al. 2016). Moreover, the microbiomes of cryopulverised root apices, including intracanal biofilms, and their paired periapical lesions in persistent apical periodontitis were compared, which revealed similar bacterial compositions (Pérez‐Carrasco et al. 2023). Only a few studies directly compared the microbiota of intracanal and matched extraradicular biofilms associated with persistent apical periodontitis (Sun et al. 2022). Comparison of microbial communities of root canal fillings and extraradicular biofilms and periapical lesions in persistent apical periodontitis revealed the presence of diverse bacteria in these sites (Sun et al. 2022). Thus, many aspects regarding the correlation between intracanal and extraradicular biofilms remain unresolved.

As bacterial biofilms around the apex are considered to play a key role in disease progression and persistence, a comprehensive analysis of apical intracanal and extraradicular biofilms may lead to a better understanding of the mechanisms underlying persistent apical periodontitis.

Therefore, in this study, we aimed to assess the characteristics of the microbiome and predicted functional pathways of extraradicular biofilm and those of the corresponding apical intracanal biofilm obtained from the same tooth with persistent apical periodontitis using next‐generation sequencing. The null hypothesis of this study was that (i) the bacterial composition is similar and (ii) there exists no difference in the predicted functional pathways between the extraradicular and paired intracanal biofilms.

## 2 Materials and Methods

### 2.1 Participant Selection

This observational study was reported under Preferred Reporting items for Observational studies in Endodontics (PROBE) 2023 guidelines. Figure S1 shows the flow chart. The ethics board reviewed and approved the study (approval number: R1‐E36, approval date: January 28, 2020), and all participants provided informed consent. Samples were collected from February 2020 to March 2023. The inclusion criteria comprised tooth being diagnosed with persistent apical periodontitis, undergoing repeated root canal treatment, exhibiting apical radiolucency despite adequate root canal obturation and satisfactory coronal restoration confirmed by radiographic and CBCT examination, and no clinical healing of periapical lesions, such as persistent pain or a non‐resolving sinus tract. Apical surgery was selected as the treatment modality instead of further non‐surgical retreatment. The exclusion criteria included teeth with periodontal pockets > 3 mm, mobility, root fractures, separated endodontic instruments, perforations or direct exposure of the root canal‐filling material to the oral cavity, and being pregnant, under 20 years old of age, or receiving antibiotics within the last 3 months. All included teeth had undergone multiple rounds of conventional endodontic treatment before surgical intervention was indicated.

### 2.2 Sample Collection and DNA Extraction

Ten of the apical samples used in this study were obtained from apicoectomies and seven from intentional replantations. All samples were strictly collected under aseptic conditions using sterile instruments and materials. For apicoectomy, a mucoperiosteal flap was elevated to expose the root apex, which was then resected in situ using a sterile diamond bur. For intentional replantation, the tooth was gently extracted using forceps, with care, to avoid contamination of the apical region, and the root apex was resected extraorally using a sterile diamond bur. In both procedures, the resected root apex was immediately washed with sterile saline to remove blood and planktonic bacteria and then immersed in RNAlater (Thermofisher scientific, Waltham, MA, USA). The periapical lesion was not included in this study. The root surfaces around the root apex were curetted with a sterile small excavator and used as extraradicular biofilm samples as previously described (Noguchi et al. 2005). No calculus was observed in any cases. The external root surfaces were disinfected with 2.5% sodium hypochlorite, followed by sodium hypochlorite inactivation using 5% sodium thiosulfate (Alves et al. 2009). To avoid potential penetration of sodium hypochlorite into the main root canal system through exposed dentinal tubules at the resected surface, the cut surface of the apex was intentionally left untreated as previously described (Pérez‐Carrasco et al. 2023; Arias‐Moliz et al. 2024). Thereafter, the root apex was frozen at −80°C until cryopulverisation. For cryopulverisation of the root apex, the sample was immersed in liquid nitrogen and homogenised using a TissueLyser (QIAGEN, Hilden, Germany), and these were used as intracanal samples. DNA was extracted using DNeasy PowerSoil Pro Kit (QIAGEN) according to the manufacturer's protocol.

### 2.3 16S rRNA Sequence Analysis

DNA libraries were prepared according to the Illumina 16S Metagenomic Sequencing Library Preparation Guide using the primer set 27Fmod (AGR GTT TGATCMTGG CTC AG) and 338R (TGC TGC CTC CCG TAG GAG T), targeting the V1–V2 region of the 16S rRNA gene. A 251‐bp paired‐end sequencing was performed using the MiSeq Reagent Kit v2 (500 cycles) and a MiSeq instrument (Illumina Inc., San Diego, CA, USA). The obtained paired‐end sequences were merged, filtered and denoised using DADA2. Taxonomic assignment of amplicon sequence variants (ASVs) was performed using the Quantitative Insights into Microbial Ecology (QIIME2) feature classifier plugin in the Greengene database (v. 13.8) and Human Oral Microbiome Database (HOMD, v. 16.02). Sequences were analysed using the QIIME2 pipeline (https://qiime2.org/). Phylogenetic Investigation of Communities by Reconstruction of Unobserved States (PICRUSt) was used to predict bacterial function from 16S rRNA gene sequencing data. Microbial functional pathways were analysed using Kyoto Encyclopaedia of Genes and Genomes (KEGG) level 3.

### 2.4 Statistical Analysis

Statistical analysis was performed, and graphical outputs were prepared using IBM SPSS Statistics (v. 22.0; IBM SPSS Inc., Endicott, NY, USA). The Wilcoxon signed‐rank sum test was used to evaluate alpha diversity. The Kruskal–Wallis test was used to evaluate beta diversity. Non‐metric multidimensional scaling (NMDS), permutational multivariate analysis of variance (PERMANOVA) and analysis of similarity (ANOSIM) based on Bray–Curtis distance were also performed using R software v.4.3.1 (R Core Team, Vienna, Austria) and the vegan package. To identify significantly different microbial genera and predicted functions between the groups, linear discriminant analysis effect size (LEfSe) analysis (*p* < 0.05) was performed. A value of *p* < 0.05 was considered statistically significant.

## 3 Results

### 3.1 Diversity of Bacterial Communities

Of the 22 samples collected, five were excluded because no apical fragment of sufficient size and morphology for cryopulverisation could be obtained during apical resection or because periodontal pockets emerged at the time of surgery despite being absent at recruitment. Consequently, 17 samples were included in the final analysis. Information on the characteristics of the participants whose samples were used for the analysis is presented in Table 1.

**TABLE 1:** Participant information.

Patient	Sex	Age (years)	Tooth	Sample ID	Surgical treatment	Spontaneous pain	Percussion pain	Palpation	Sinus tract
1	F	55	Upper premolar	A	A		+		
2	F	45	Upper incisor	B	A				
3	M	69	Upper molar	C	A			+	+
			Upper molar	D	A			+	+
4	F	67	Lower molar	E	A			+	+
			Lower molar	F	A			+	+
5	M	42	Lower premolar	G	R		+		+
6	F	49	Upper molar	H	A	+	+		
7	F	67	Upper incisor	I	A			+	+
8	M	51	Upper molar	J	R	+	+	+	
9	F	47	Upper molar	K	R	+	+		
10	F	63	Lower molar	L	R	+	+	+	
11	M	75	Upper molar	M	R		+	+	
			Upper molar	N	R		+	+	
			Upper molar	O	R		+	+	
12	M	56	Upper premolar	P	A				+
13	F	55	Lower molar	Q	A				+

*Note:* Surgical treatment: A = apicoectomy, R = intentional replantation.

**FIGURE 1:**
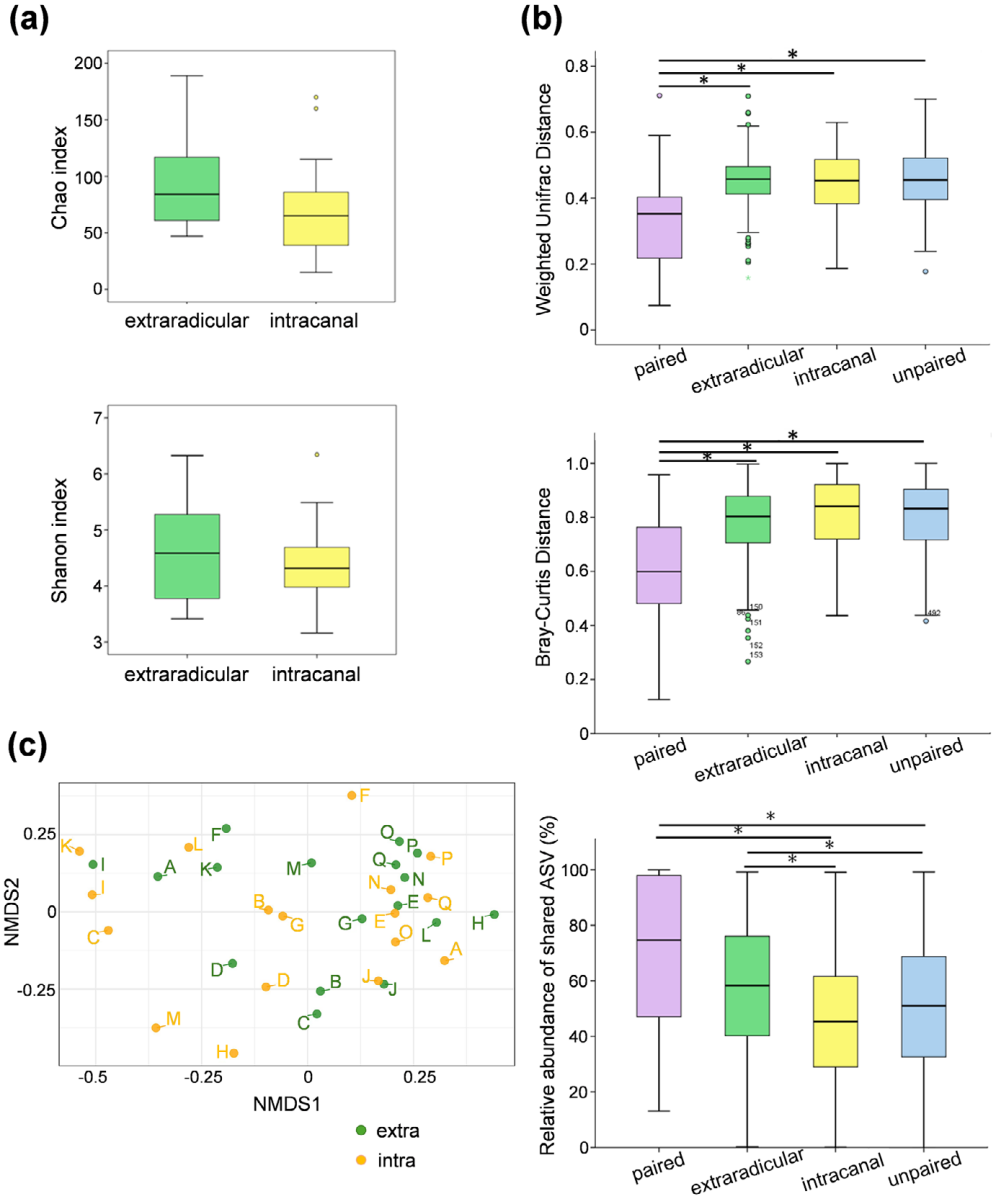
Alpha and beta diversity indices of the microbial communities. (a) Chao and Shannon indices for extraradicular and intracanal biofilms. (b) Comparison of weighted UniFrac, Bray–Curtis distances and the relative abundance of shared ASVs between paired extraradicular and intracanal biofilms, extraradicular biofilms, intraradicular biofilms, and unpaired extraradicular and intracanal biofilms. *Significant differences between compared groups (*p* < 0.05). (c) Non‐metric multidimensional scaling (NMDS) analysis of extraradicular and intracanal biofilm samples. Each letter next to a dot represents a sample ID. Next to the dot, extra = extraradicular biofilm, intra = intracanal biofilm.

Sequencing yielded a total of 1 713 717 reads, with an average of 50 403 reads per sample. Microbiome alpha diversity analysis revealed that the Chao and Shannon indices were not significantly different between the two sampling sites (Figure 1a). In contrast, beta diversity analysis showed that microbial communities in paired samples exhibited significantly lower weighted UniFrac and Bray–Curtis distances of samples from the same root canal system than those between unpaired samples (Figure 1b). The unweighted UniFrac and Jaccard distances were also significantly lower in paired samples compared to unpaired samples (Figure S2). The community composition between the two sites was not clearly distinguished by NMDS analysis (Figure 1c; PERMANOVA, *p* = 0.666; ANOSIM, *R* = −0.013, *p* = 0.578). PERMANOVA revealed no statistically significant differences between paired extraradicular and intracanal biofilms, regardless of the presence of the sinus tract or the surgical method used (Figure S3). No significant interactions were found between sample site (intracanal/extraradicular) and either sinus tract presence or the surgical method used (sinus tract, *p* = 0.854; surgical method, *p* = 0.820). The relative abundance of shared ASVs between paired samples was significantly lower than between unpaired samples (Figure 1b).

**FIGURE 2:**
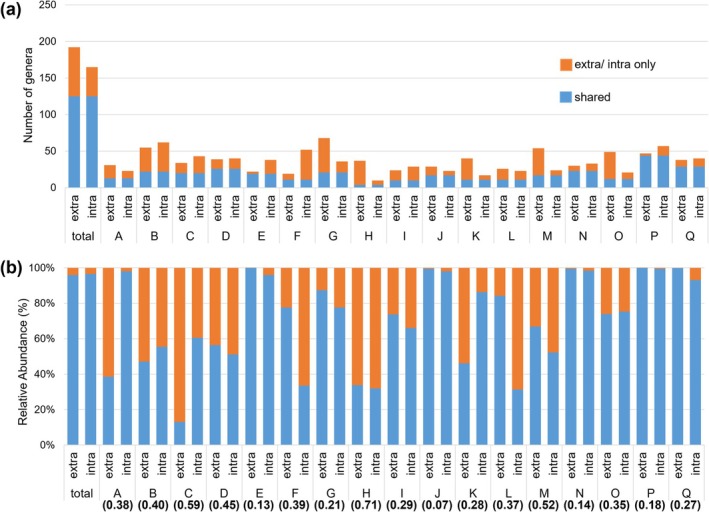
Prevalence of shared and unshared bacterial composition at the genus level. (a) Number of shared or unshared genera detected in the samples. (b) Relative abundance of shared or unshared genera across samples. The far left represents all bacterial composition detected in samples A–Q. extra = extraradicular biofilm, intra = intracanal biofilm. The numbers in parentheses below the sample name indicate weighted UniFrac distance.

Figure 2 shows the number of shared and unshared genera detected in the samples and their relative abundances at the genus level. Although individual differences were observed between samples, 125 shared genera were identified across all samples, accounting for 65.1% of the total genera found in extraradicular biofilms and 75.8% in intracanal biofilms. The number of unshared species was higher in extraradicular biofilms than in intraradicular biofilms, with averages of shared relative abundances being 95.9% and 96.6%, respectively. The number of shared species and their relative abundance did not always coincide, with some samples sharing fewer species but exhibiting a higher abundance, whereas others shared a lower number and also had a lower abundance.

### 3.2 Composition of Bacterial Communities

**FIGURE 3:**
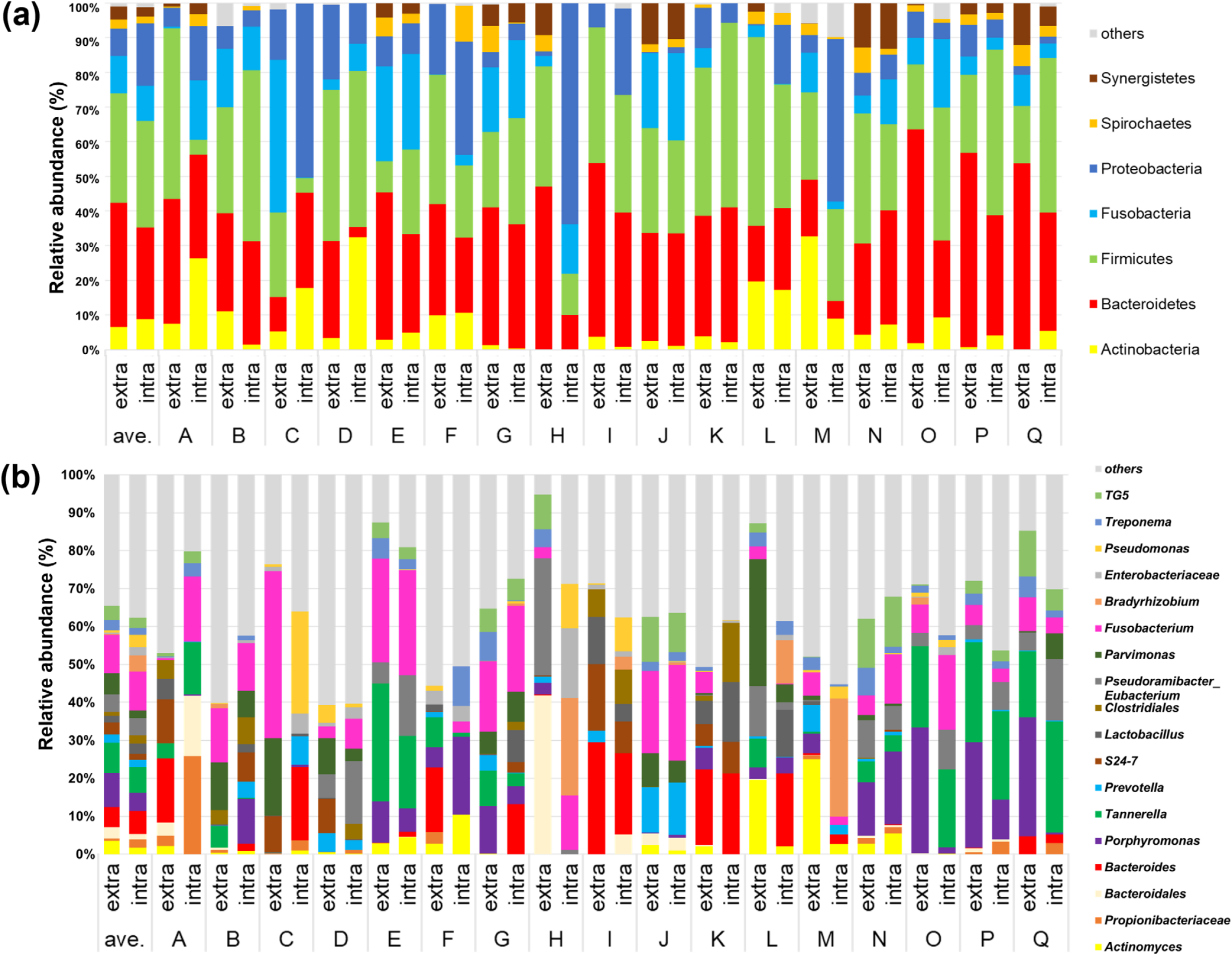
Relative abundance of bacterial phyla (a) and genera (b) in each extraradicular and intracanal biofilm sample and average of all samples. ave. = average of all samples, extra = extraradicular biofilm, intra = intracanal biofilm.

The composition of the bacterial taxa at the phylum level in the biofilm samples is shown in Figure 3a. Biofilm‐forming bacteria belonged to 14 bacterial phyla. On average, for all samples, 99% of the taxa belonged to seven phyla. Using the Greengenes database, Bacteroides, Firmicutes, Fusobacteria and Proteobacteria were the dominant phyla, with average relative abundances of 35.8%, 31.6%, 10.7% and 7.8% in extraradicular biofilms and 30.7%, 26.4%, 10.2% and 18.1% in intraradicular biofilms, respectively. Although slight differences in relative abundances were observed, these phyla remained among the most dominant groups when classified using both SILVA and HOMD databases (Figure S4). Figure 3b shows the composition of the bacterial taxa in the biofilm samples at the genus level. In extraradicular biofilms, *Fusobacterium* (10.3%) was the most abundant, followed by *Porphyromonas* (9%), *Tannerella* (8%), *Parvimonas* (5.5%) and *Bacteroides* (5.3%). In intracanal samples, *Fusobacterium* (10.2%) was the most abundant, followed by *Tannerella* (6.7%), *Bacteroides* (6.1%), *Porphyromonas* (4.8%) and *Pseudoramibacter‐Eubacterium* (4.5%). These genera were also dominant when classified using other databases, although their relative order differed (Figure S4).

**FIGURE 4:**
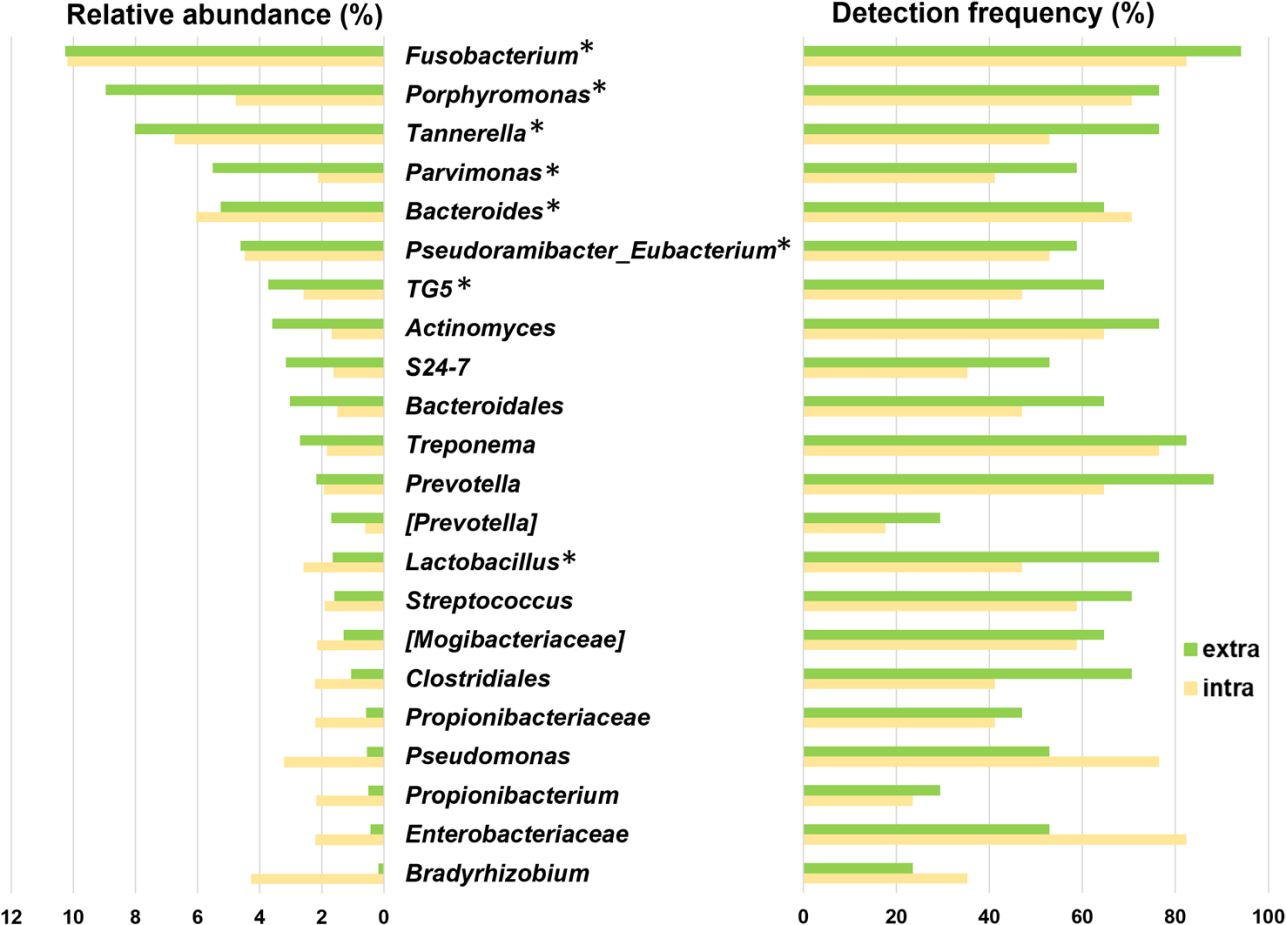
Average relative abundance of the top 15 most abundant bacterial genera in each biofilm sample (left), and their detection frequency across samples (right). Asterisk represents genera that ranked within the top 15 in both samples. extra = extraradicular biofilm, intra = intracanal biofilm.

The top 15 bacterial genera in each biofilm sample, based on average relative abundance, and their detection frequencies across the samples are shown in Figure 4. A total of 22 genera were identified from the combined top 15 lists of the intracanal and extraradicular biofilms, among which eight genera were shared between both groups. Notably, the highest ranking genera tended to overlap, with the top 7 genera being identical in both biofilm types. No statistical differences were found in the relative abundances and detection frequencies of the 22 genera between the intracanal and extraradicular biofilms. *Fusobacterium* was the most abundant species detected at both sites. Obligate anaerobes, such as *Treponema*, *Prevotella*, *Porphyromonas* and *Bacteroides* as well as gram‐positive bacteria, including *Actinomyces*, were highly detected (in more than 60% of the samples) at both sides. Although the choice of reference database influenced the bacterial composition, the top six genera were consistently identified across all three databases. Furthermore, genera commonly found in both biofilm groups tended to rank higher in abundance, indicating a degree of biological consistency across sampling sites (Figure S5).

**FIGURE 5:**
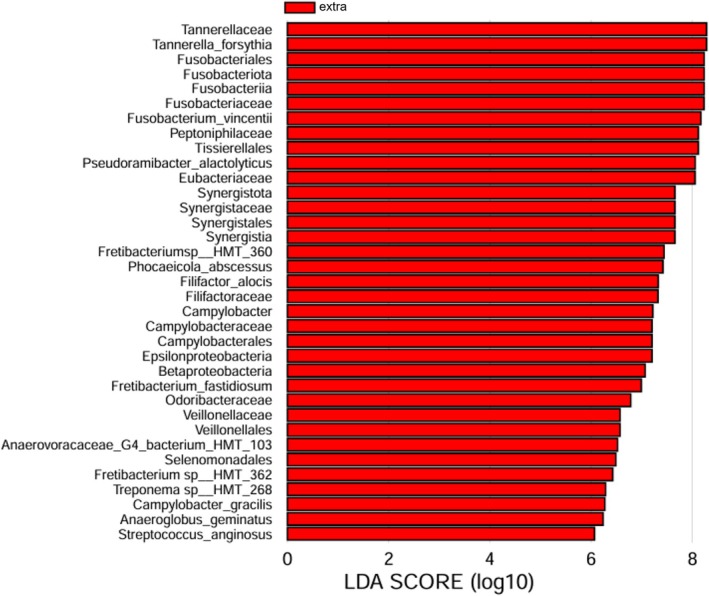
Genera with significant differences between groups as determined by linear discriminant analysis effect size (LEfSe) analysis. LDA > 6, *p* < 0.05, extra = extraradicular biofilm, intra = intracanal biofilm.

LEfSe analysis was conducted to identify differences between the groups using HOMD at the species level (Figure 5). Despite the non‐significant results from PERMANOVA and ANOSIM, LEfSe analysis showed that 35 bacterial taxa, including 12 species, were significantly more abundant in extraradicular, whereas no taxa were significantly enriched in intraradicular biofilms. Of these 12 species, six, including *Tannerella forsythia* , *Fusobacterium vincentii*, *Pseudoramibacter alactolyticus* , *Phocaeicola abscessus* , *Campylobacter gracilis* and *Fretibacterium* sp. HMT 360, showed relative proportions > 1%, while the remaining six species had relative proportions < 1%.

### 3.3 Predictive Analysis of Bacterial Function

**FIGURE 6:**
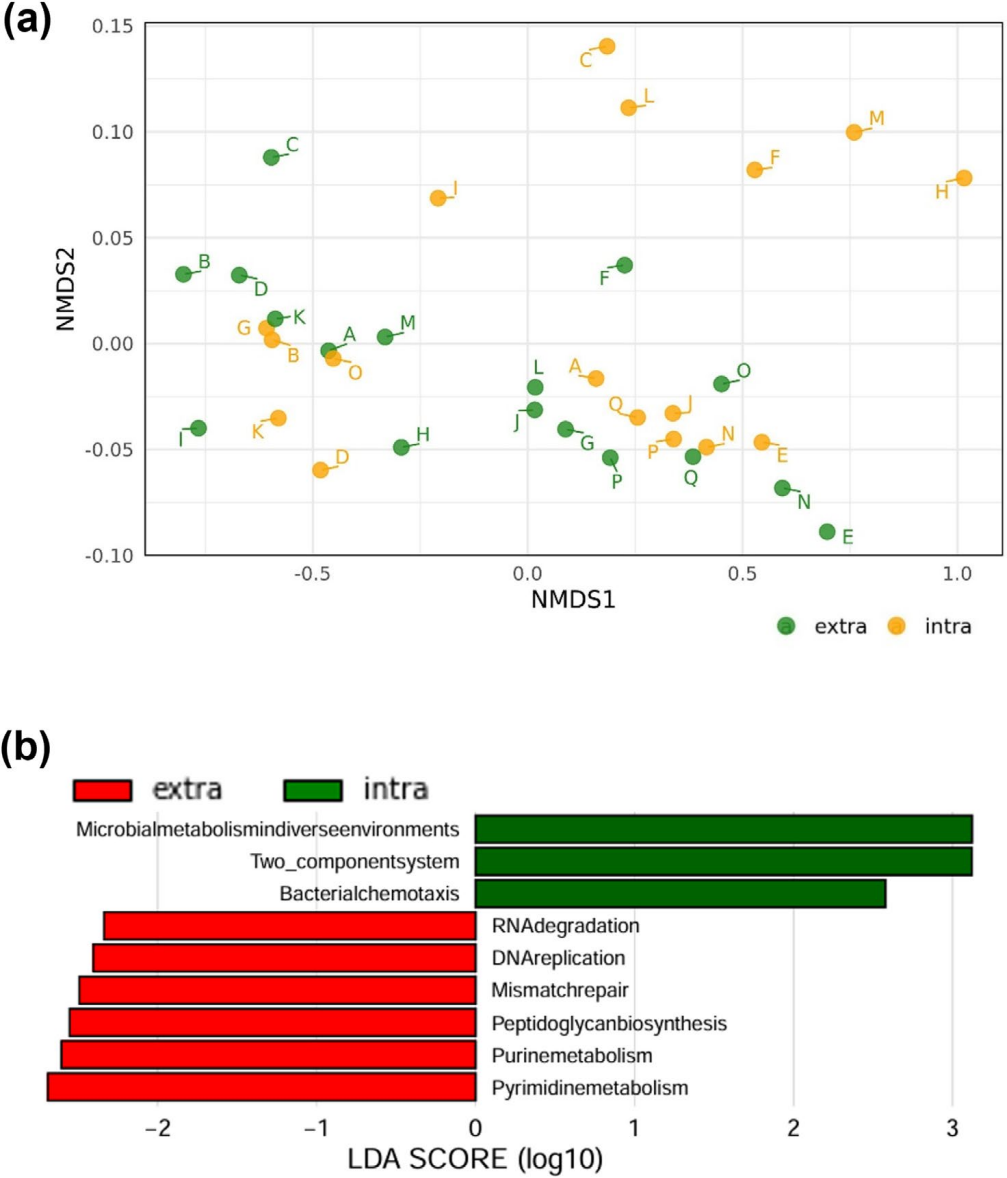
Predictive functional analysis of the biofilm samples. (a) Non‐metric multidimensional scaling analysis based on Bray–Curtis distance of the intracanal and extraradicular biofilm samples. (b) Linear discriminant analysis effect size analysis of Kyoto Encyclopaedia of Genes and Genomes pathways at level 3. LDA > 2, *p* < 0.05, extra = extraradicular biofilm, intra = intracanal biofilm.

Although the NMDS analysis (Figure 6a; PERANOVA, *p* = 0.219) did not clearly distinguish the predicted bacterial functions between the two sites, several KEGG pathway differences were observed between extraradicular and intraradicular biofilms (Figure 6b). Several pathways related to nucleotide metabolism, peptidoglycan biosynthesis, genetic replication and repair or translation were enriched in extraradicular biofilms. In contrast, in the intraradicular biofilms, a few pathways related to cell motility and signal transduction were significantly enriched.

## 4 Discussion

In this study, we compared the bacterial compositions of extraradicular and paired intracanal biofilms. Weighted UniFrac and Bray–Curtis distances between paired samples from the same root were significantly lower than those between samples from different roots, indicating greater similarity in bacterial composition within the same root. Consistently, the relative abundance of shared ASVs was significantly higher in paired samples (median: 74.7%) than in intracanal (median: 45.3%) or unpaired samples (median: 51%), suggesting that extraradicular biofilm bacteria are usually likely derived from intracanal microbiota. The relative abundance of shared ASVs among extraradicular biofilms (median: 58.3%) was also significantly higher than that of intracanal biofilms and unpaired samples; this may be supported by nutrient supply from tissue fluids and inflammatory exudates via the apical foramen and by reduced inter‐individual environmental variation (Siqueira et al. 2024).

Although bacterial invasion via sinus tracts could potentially influence extraradicular microbiota, no significant differences in beta diversity were observed between samples with and without sinus tracts, suggesting a limited impact on microbial similarity. Consistent with our results, Schuweiler et al. (2024) reported no significant effect of sinus tracts on microbial composition based on amplicon sequencing of endodontic samples from 71 patients. In contrast, Sun et al. (2022) reported differences in bacterial composition between endodontic infections with and without sinus tracts. Moreover, studies utilising checkerboard DNA–DNA hybridisation have shown contradictory findings, with one study reporting the presence of taxa significantly associated with sinus tracts (Sassone et al. 2008) and another finding no evidence of such specificity (Rôças et al. 2011). Such discrepancies indicate that additional factors, such as methodological differences, sampling sites, infection stage and host environment, may also influence bacterial composition associated with sinus tracts.

Considerable variation was observed in bacterial community similarity across samples. For the Bray–Curtis and weighted UniFrac distances, 4 of the 17 paired samples (23.5%) were above the overall average, indicating a more distinct bacterial community. One sample had a lower number of shared genera and relative abundance in both intracanal and extraradicular biofilms, suggesting a large environmental difference or alternative infection routes. While environmental differences and the mechanisms by which biofilms form on extraradicular surfaces may influence the similarity of bacterial composition between intracanal and extraradicular biofilms, the presence or absence of the sinus tract did not affect the similarity in this study.

NMDS analysis revealed no significant differences in microbial diversity between sites, supporting the first null hypothesis. Sun et al. (2022) reported that curetted root canal fillings and matched extraradicular biofilms had similar microbial profiles. The analysis herein included both the contents of the root canal and bacteria that had infiltrated the dentine tubules owing to freeze pulverisation and consistently demonstrated similarities between the microbiota of the intracanal and extraradicular biofilms. The predominant genera in the intracanal included *Fusobacterium, Tannerella*, *Bacteroides*, *Porphyromonas* and *Pseudoramibacter‐Eubacterium*, consistent with previous NGS studies evaluating the apical microbiota of post‐treated infections (Siqueira et al. 2016; Bouillaguet et al. 2018; Pérez‐Carrasco et al. 2023). Additionally, *Fusobacterium, Porphyromonas*, *Tannerella*, *Parvimonas* and *Bacteroides* have been frequently detected in extraradicular biofilms (Noguchi et al. 2005; Narayanan and Vaishnavi 2010). These taxa, along with *Actinomyces*, *Prevotella* and *Treponema*—which were predominant in extraradicular biofilm—are also common in periapical lesions (Bronzato et al. 2021), suggesting microbial similarity across intracanal, extraradicular and periapical sites. Notably, *Porphyromonas gingivalis* facilitates biofilm persistence by escaping host immune responses through various virulence factors (Zheng et al. 2021) and protecting *Tannerella forsythia* and *Fusobacterium nucleatum* from neutrophil‐mediated killing (Angabo et al. 2024). As a result, bacteria capable of immune evasion and their beneficiaries are more likely to persist and proliferate.

While the overall intracanal and extraradicular microbiota were similar, several taxa enriched in extraradicular biofilms may contribute to pathogenesis through interspecies interactions. *F. vincentii* functions as a bridging organism for colonisation by diverse anaerobic bacteria (Yonezawa et al. 2024), while *T. forsythia* interacts with neighbouring species through adhesins, enhancing polymicrobial virulence (Inagaki et al. 2005). Members of Veillonellaceae utilise lactate produced by early colonisers, such as *Streptococcus* (Mashima and Nakazawa 2014), promoting the growth of late anaerobes, including *Prevotella* and *Fusobacterium*, and supporting biofilm development (Zhou et al. 2021). *F. alocis* likely engages in syntrophic amino acid degradation and cooperates with *P. gingivalis* to enhance biofilm persistence, virulence and oxidative stress resistance (Aruni et al. 2015). Similarly, the asaccharolytic fastidious anaerobe *Fretibacterium* spp. may depend on metabolic complementation with proteolytic species, such as *Fusobacterium*, to persist in advanced oral infections (Vartoukian et al. 2013). Additionally, *C. gracilis* , a nitrate‐reducing anaerobe, may support microbial persistence via metabolic cross‐feeding (D'Souza et al. 2018), while epithelial invasion and immune modulation by *Campylobacter* spp. may indicate broader survival strategies (Kemper and Hensel 2023). Such metabolic mutualism and inter‐bacterial interactions may contribute to biofilm maturation, stability and persistence under inflammatory and nutrient‐limited conditions (Miller et al. 2019).

We also compared the predicted functional pathways of extraradicular and intracanal biofilms to examine their functional roles. The second null hypothesis was partially rejected; although PERMANOVA showed no significant differences in the overall microbial structure, LEfSe analysis indicated subtle but potentially relevant differences in the predicted metabolic pathways. In extraradicular samples, pathways related to nucleobase metabolism, including purine and pyrimidine metabolism, were enriched, suggesting enhanced bacterial growth, biofilm formation and virulence potential. Purine metabolism is essential for bacterial proliferation and biofilm formation and has been linked to virulence in *Staphylococcus aureus* and *Enterococcus faecalis* (Gélinas et al. 2021; Goncheva et al. 2019). Similarly, pyrimidine biosynthesis plays a role in bacterial growth, survival in macrophages, biofilm formation and virulence factor production (Garavaglia et al. 2012; Rossi et al. 2022). Increased nucleotide metabolism has also been implicated in stress adaptation and survival in *E. faecalis* (Ran et al. 2015), suggesting potential roles in bacterial adaptation and survival under external stress. While direct evidence for nucleotide‐based cross‐feeding among oral bacteria remains limited, comparable interactions in other microbial systems support coexistence under nutrient‐restricted environments (D'Souza et al. 2018). Moreover, enrichment of peptidoglycan biosynthesis pathways, which contribute to bacterial cell protection, growth, division and antibiotic resistance (Cui et al. 2003; Whitley et al. 2024), may further support bacterial persistence under host‐ and treatment‐related stressors. These observations indicate that extraradicular persistence may be driven by functional adaptations rather than compositional differences alone.

In contrast, intraradicular biofilms were characterised by enrichment of pathways related to bacterial chemotaxis and signal transduction. Bacterial chemotaxis, a key component of motility, enables migration towards favourable niches (Matilla and Krell 2018) and is frequently observed in endodontic infections, including *F. nucleatum* (Mansour 2023). In parallel, the two‐component system (TCS), a signal transduction pathway regulating stress responses, virulence and biofilm formation (Shaw et al. 2022), was also enriched. TCS enables bacteria to respond to physicochemical fluctuations (Tiwari et al. 2017), which may facilitate adaptive survival under dynamically changing conditions within intraradicular biofilms. Since chemotaxis is frequently TCS regulated, enhanced TCS activity may allow bacteria to translate environmental cues into motility and adaptive behaviours (Matilla and Krell 2018). In nutrient‐limited root canals, chemotaxis may optimise spatial positioning and support persistence, while also facilitating extraradicular invasion by detecting host‐derived chemoattractants, allowing escape from adverse intracanal conditions and colonisation of nutrient‐rich niches, contributing to host evasion and treatment resistance (Siqueira and Rôças 2007). These suggest that specific functions may vary between groups even when the overall composition is similar.

Both apicoectomy and intentional replantation were included in this study to enhance the anatomical diversity of the sampled sites, as intentional replantation is often selected when apicoectomy is anatomically challenging (Plotino et al. 2022). This approach enabled sampling from a broader range of tooth positions, improving the generalisability of our microbial findings. Although intentional replantation may pose a higher risk of sampling‐related contamination, the meticulous sampling procedure and thorough washing of adherent planktonic bacteria ensured sample reliability. Therefore, PERMANOVA based on the Bray–Curtis distance revealed no significant differences between samples obtained via the two procedures, suggesting comparative bacterial community structure and composition.

Nonetheless, the study has some limitations. Despite differences in taxonomic resolution among reference databases, the top six bacterial genera were consistently identified across Greengenes, SILVA and HOMD, suggesting that dominant taxa in root‐associated biofilms are robustly detected regardless of the classification framework. However, database selection can substantially influence the resolution of less dominant taxa. Thus, to enhance clinical relevance, we reanalysed the data using HOMD at the species level and provided supplemental genus‐level comparisons using SILVA and HOMD. Widely used databases, such as Greengenes, may underestimate or misclassify oral taxa and hinder cross‐study comparisons (Nagai et al. 2024). Although efforts have been made to evaluate optimal databases for oral microbiome analysis (Nagai et al. 2024), the lack of standardised criteria for database selection and curation remains a recognised challenge and should be considered when interpreting 16S rRNA–based taxonomic profiles. Moreover, amplicon sequencing lacks resolution below the species level, and the PICRUSt provides only predicted functional profiles. Shotgun metagenomic sequencing is a suitable approach for addressing this challenge. Furthermore, considering that viable bacteria are primarily responsible for various functions, metatranscriptomic and metaproteomic analyses offer more direct insights through transcript‐ and protein‐level evidence. Such approaches may ultimately facilitate the development of therapeutic strategies targeting bacterial functions or properties rather than specific taxa in persistent endodontic infections.

Recently, novel approaches, including narrow‐spectrum antimicrobials, such as antitoxins, bacteriophages and antibody‐conjugated drugs, have been suggested for the treatment of biofilm‐associated infections (Hwang et al. 2025). Several bacteriophages have demonstrated inhibitory effects against *F. nucleatum* (Wang et al. 2022) and antibacterial activity against *Enterococcus faecalis* , also improving periapical lesions (Kuong et al. 2025). Nanostructured antimicrobial delivery systems and synthetic antimicrobial peptides have also demonstrated inhibitory effects on major endodontic pathogens and biofilms (Xie et al. 2025; Liu et al. 2025). Moreover, functional enrichment in nucleotide metabolism suggests potential therapeutic targets, as purine metabolites regulate host immunity and antibiotic response (Carfrae and Brown 2023). Small‐molecule inhibitors targeting the TCS have demonstrated encouraging antibacterial efficacy (Ji et al. 2024). These approaches may benefit patients with persistent apical periodontitis who are not candidates for surgical treatment. By characterising difficult‐to‐eliminate bacterial communities around the apical foramen, the present study may help guide the selection of microbial and functional targets for personalised management of endodontic infections.

## 5 Conclusion

Although the bacterial composition of extraradicular and intracanal biofilms showed some differences, the two communities were broadly similar in diversity and dominant taxa. In particular, they shared many of the dominant bacterial species, indicating that the intracanal microbiota at the apices is closely associated with that of extraradicular biofilms, despite some exceptions. In addition, some differences in predicted functional profiles were identified between the two biofilm types.

## Author Contributions

**Yoko Asahi:** conceptualisation, writing – original draft, visualisation, validation, software, resources, project administration, methodology, investigation, funding acquisition, formal analysis. **Nanako Kuriki:** sample collection, methodology, investigation, writing – original draft. **Motoki Okamoto:** sample collection, investigation, writing – original draft. **Daisuke Motooka:** data curation, investigation, writing – review and editing. **Shota Nakamura:** data curation, investigation, writing – review and editing. **Hazuki Maezono:** investigation, writing – review and editing. **Kittipit Klanliang:** formal analysis, visualisation, investigation, writing – review and editing. **Tsuyoshi Shimaoka:** sample collection, investigation, writing – review and editing. **Tetsuya Iida:** investigation, writing – review and editing. **Shigeyuki Ebisu:** supervision. **Yuichiro Noiri:** investigation, writing – review and editing. **Mikako Hayashi:** investigation, writing – review and editing. All authors approved the final manuscript.

## Funding

This work was supported by Japan Society for the Promotion of Science (JP23K09198, JP20K09953).

## Ethics Statement

This study was approved by the Ethics Committee of Osaka University Graduate School of Dentistry (R1‐E36, January 28, 2020).

## Consent

Informed consent was obtained from all participants prior to their inclusion in the study, and samples were collected in accordance with the Helsinki Declaration.

## Conflicts of Interest

The authors declare no conflicts of interest.

## Supporting information


**Figure S1:** Flow chart of the samples for this study.

**Figure S2:** Comparison of beta diversity among different sites.

**Figure S3:** Non‐metric multidimensional scaling analysis based on different conditions.

**Figure S4:** Relative abundance of bacteria. Phyla level: (a) SILVA, (b) HOMD and genus level: (c) SILVA, (d) HOMD. Taxonomic labels reflect the original phylum names used in each reference database.

**Figure S5:** Relative abundance of the top 15 most abundant bacterial genera in each group using SILVA (a) and HOMD (b) databases. Relative abundance in each biofilm (left), and their detection frequency within patients (right). Asterisk represents genera that ranked within the top 15 in both extraradicular (extra) and intracanal (intra) biofilms.

## Data Availability

The data that support the findings of this study are available from the corresponding author upon reasonable request.
