# Supplementary figures and images for: Microbiome of Apical Intracanal and Extraradicular Biofilms From the Same Roots of Teeth With Persistent Apical Periodontitis: An Observational Study
Source: Int Endod J. 2026 Feb 11;59(5):806–18. doi: 10.1111/iej.70113 (PMC13065889; doi:10.1111/iej.70113)

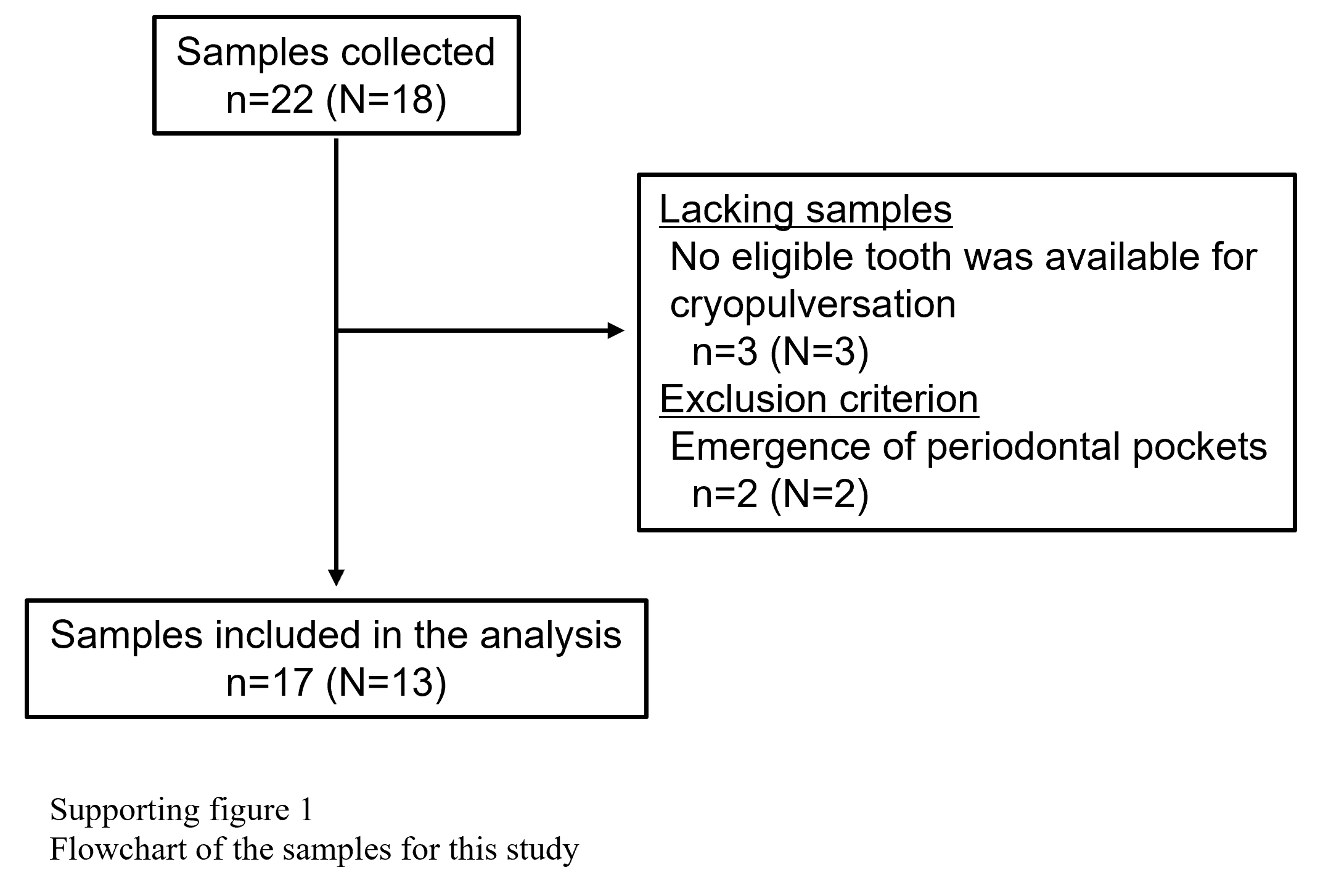

Supplement: Supplementary file 1 — Figure S1: Flow chart of the samples for this study. [file IEJ-59-806-s002.tif]

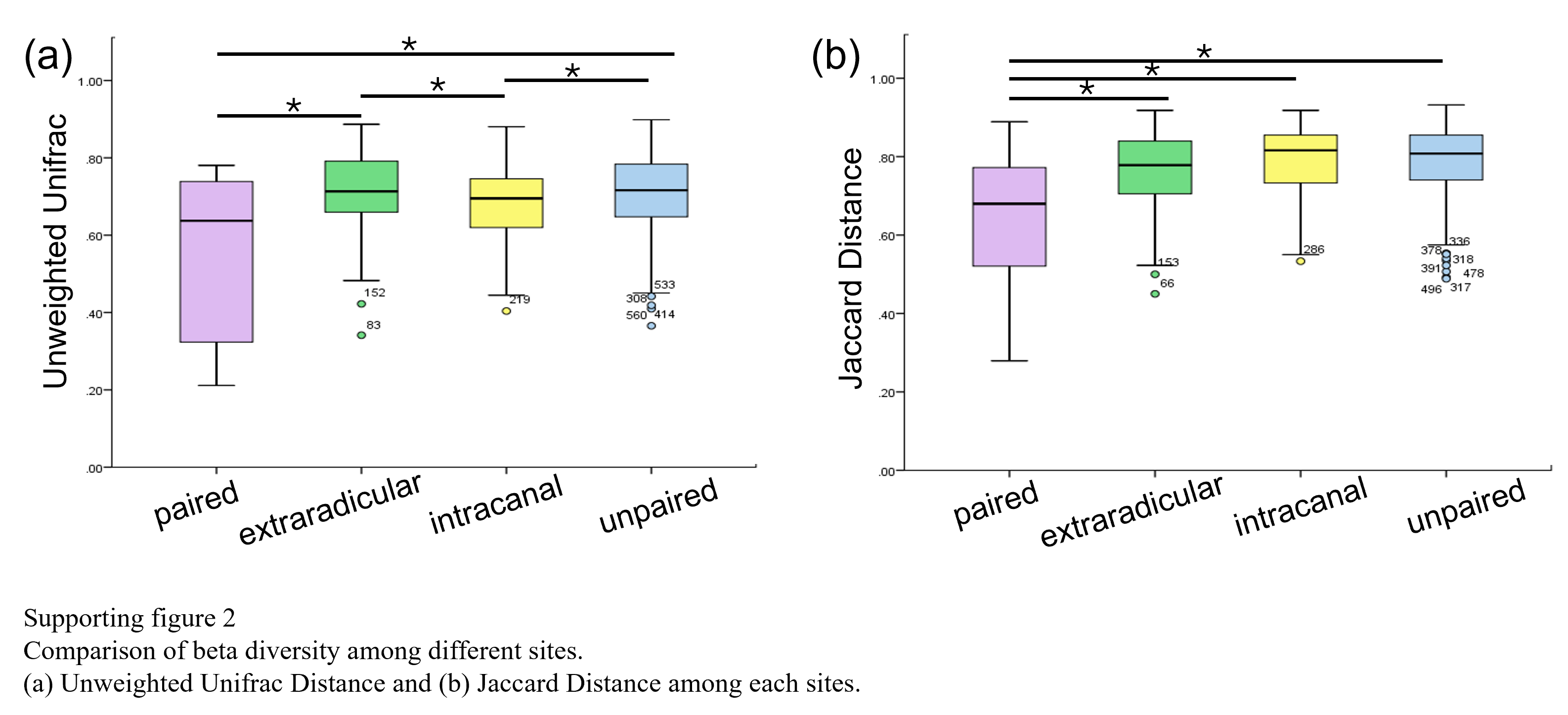

Supplement: Supplementary file 2 — Figure S2: Comparison of beta diversity among different sites. [file IEJ-59-806-s003.tif]

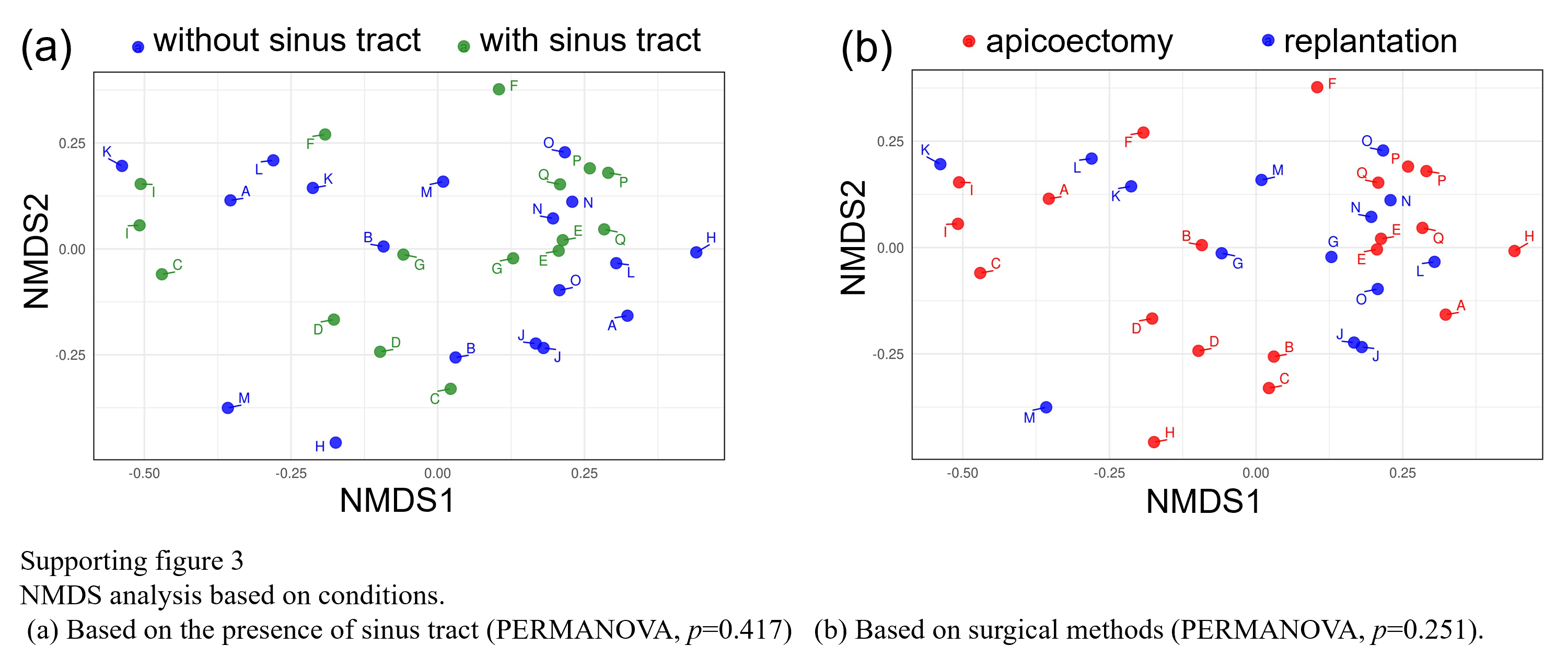

Supplement: Supplementary file 3 — Figure S3: Non‐metric multidimensional scaling analysis based on different conditions. [file IEJ-59-806-s005.tif]

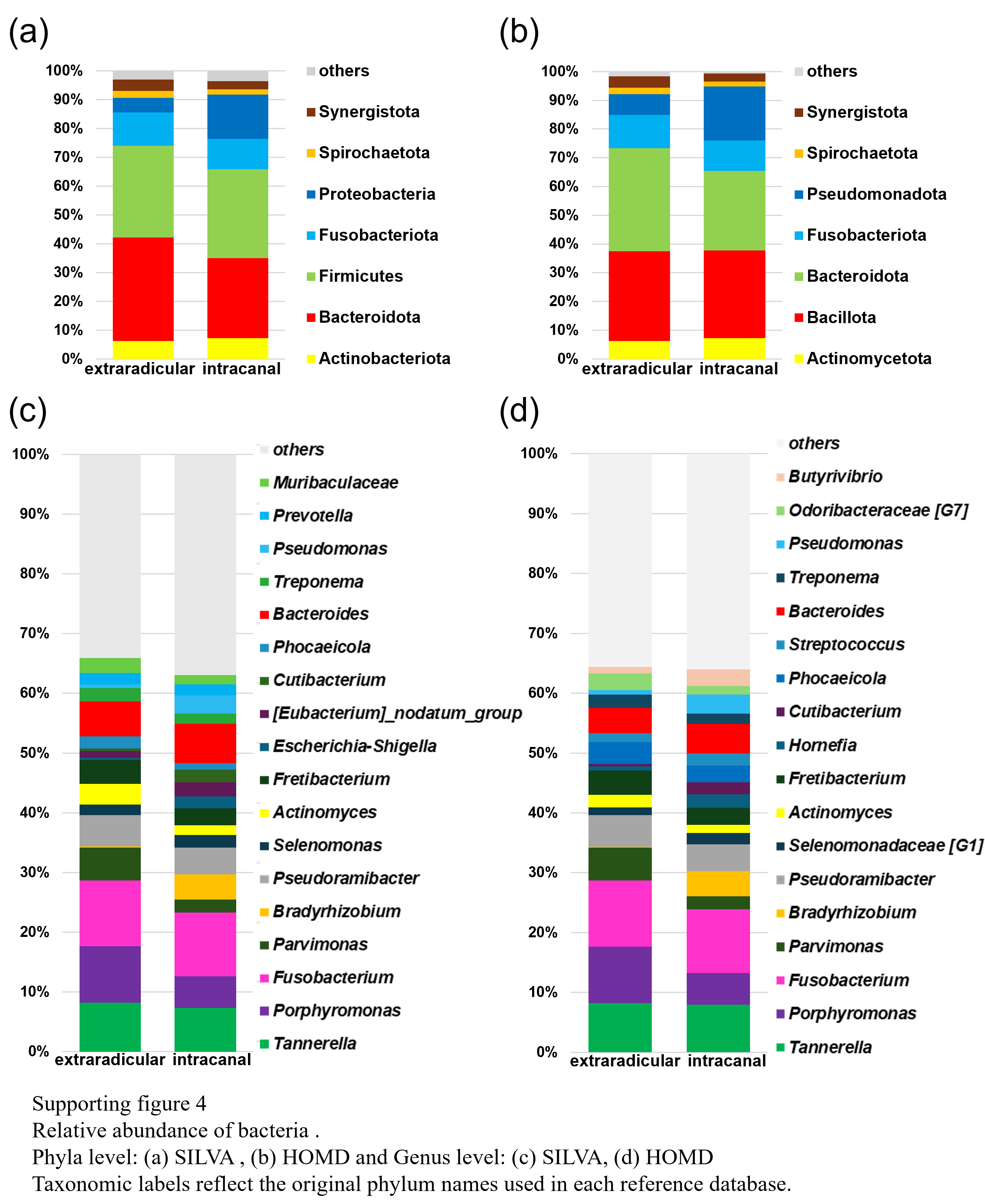

Supplement: Supplementary file 4 — Figure S4: Relative abundance of bacteria. Phyla level: (a) SILVA, (b) HOMD and genus level: (c) SILVA, (d) HOMD. Taxonomic labels reflect the original phylum names used in each reference database. [file IEJ-59-806-s004.tif]

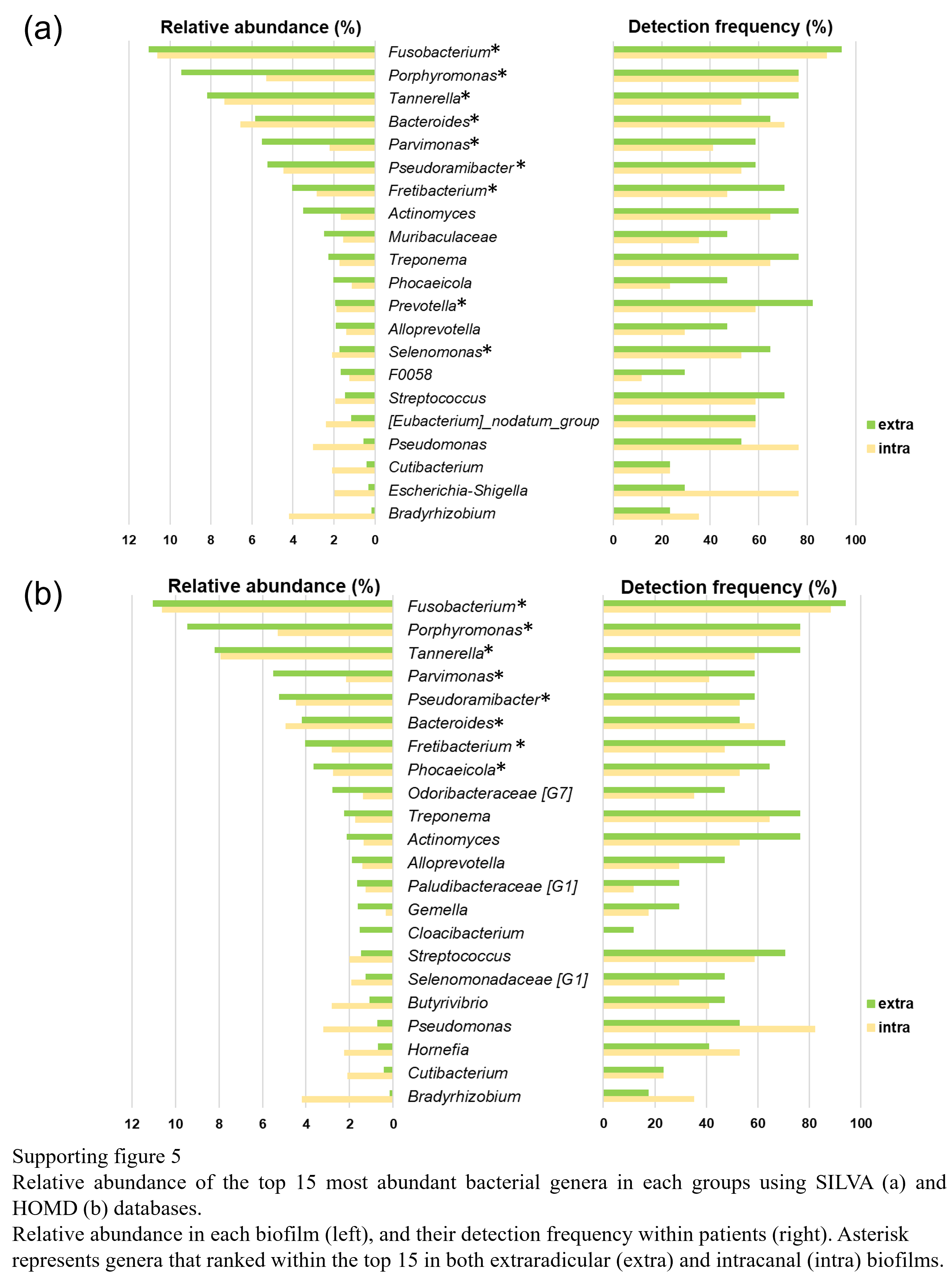

Supplement: Supplementary file 5 — Figure S5: Relative abundance of the top 15 most abundant bacterial genera in each group using SILVA (a) and HOMD (b) databases. Relative abundance in each biofilm (left), and their detection frequency within patients (right). Asterisk represents genera that ranked within the top 15 in both extraradicular (extra) and intracanal (intra) biofilms. [file IEJ-59-806-s001.tif]
